# Hereditary Hearing Impairment with Cutaneous Abnormalities

**DOI:** 10.3390/genes12010043

**Published:** 2020-12-30

**Authors:** Tung-Lin Lee, Pei-Hsuan Lin, Pei-Lung Chen, Jin-Bon Hong, Chen-Chi Wu

**Affiliations:** 1 Department of Medical Education, National Taiwan University Hospital, Taipei City 100, Taiwan; tyerainforest@gmail.com; 2 Department of Otolaryngology, National Taiwan University Hospital, Taipei 11556, Taiwan; ru3au3@gmail.com; 3 Graduate Institute of Clinical Medicine, National Taiwan University College of Medicine, Taipei City 100, Taiwan; paylong@ntu.edu.tw; 4 Graduate Institute of Medical Genomics and Proteomics, National Taiwan University College of Medicine, Taipei City 100, Taiwan; 5 Department of Medical Genetics, National Taiwan University Hospital, Taipei 10041, Taiwan; 6 Department of Internal Medicine, National Taiwan University Hospital, Taipei 10041, Taiwan; 7 Department of Dermatology, National Taiwan University Hospital, Taipei City 100, Taiwan; 8 Department of Medical Research, National Taiwan University Biomedical Park Hospital, Hsinchu City 300, Taiwan

**Keywords:** syndromic hereditary hearing impairment, cutaneous abnormalities, genetic diagnosis, precision medicine

## Abstract

Syndromic hereditary hearing impairment (HHI) is a clinically and etiologically diverse condition that has a profound influence on affected individuals and their families. As cutaneous findings are more apparent than hearing-related symptoms to clinicians and, more importantly, to caregivers of affected infants and young individuals, establishing a correlation map of skin manifestations and their underlying genetic causes is key to early identification and diagnosis of syndromic HHI. In this article, we performed a comprehensive PubMed database search on syndromic HHI with cutaneous abnormalities, and reviewed a total of 260 relevant publications. Our in-depth analyses revealed that the cutaneous manifestations associated with HHI could be classified into three categories: pigment, hyperkeratosis/nail, and connective tissue disorders, with each category involving distinct molecular pathogenesis mechanisms. This outline could help clinicians and researchers build a clear atlas regarding the phenotypic features and pathogenetic mechanisms of syndromic HHI with cutaneous abnormalities, and facilitate clinical and molecular diagnoses of these conditions.

## 1. Introduction

Sensorineural hearing impairment (SNHI) is the most common form of inherited sensory defect, which occurs in approximately 1.9/1000 live births [1]. More than 50% of SNHI cases in children can be attributed to genetic causes, and are classified as hereditary hearing impairment (HHI) [2]. Over the past two decades, the genetic causes of HHI have been decoded rapidly, especially with the advent of next-generation sequencing (http://hereditaryhearingloss.org) [3]. Among the deafness genes known, some are associated with syndromic HHI, with symptoms in organ systems outside the auditory pathway. Patients suffering from various forms of syndromic HHI additionally present with skin abnormalities. The goals of this review were to perform a literature survey on comprehensive animal and human studies and to outline the molecular mechanisms underlying HHI with cutaneous abnormalities.

## 2. Materials and Methods 

**Figure 1:**
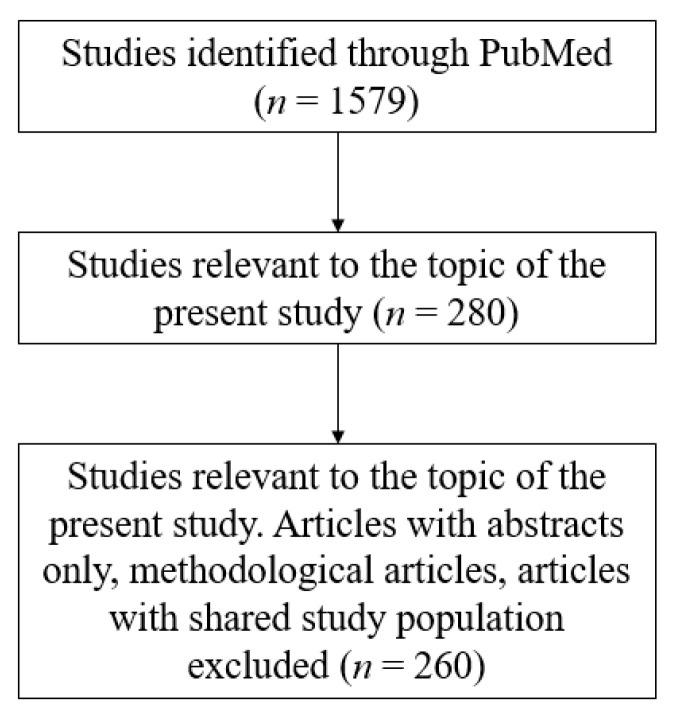
Article selection.

Our search strategy was based on using Online Mendelian Inheritance in Man (OMIM) and PubMed databases for retrieval of suitable articles relevant to our topic of interest. A collection of these publications was stored and managed on EndNote X9 (Thomson Reuters, New York City, NY, USA). Publications were eligible only if they were relevant to HHI associated with cutaneous abnormalities. Affected patients included in case reports or series were considered to be of interest only if relevant phenotypes, including abnormal cutaneous, hair, or nail findings, as well as SNHI were observed. Publications focusing on individuals with HHI and developmental disorders (e.g., distinctive facial characteristics, congenital heart defect, developmental delay, kyphosis, among others), who did not present with abnormal skin, hair, or nail findings, were not included for discussion in the present review. Studies in which the subjects discussed presented with abnormal cutaneous findings due to other proven diseases (e.g., acanthosis nigricans due to diabetes mellitus) were also excluded. A flowchart of the search strategy is shown in Figure 1.

## 3. Various Types of HHI Present with Cutaneous Abnormalities

### 3.1. Search Results

Forty-eight entries in the OMIM database with distinct “MIM (Mendelian Inheritance in Man) numbers” were selected, and a total of 260 publications were retrieved from the PubMed database, including original articles (*n* = 154), case reports (*n* = 74), and literature reviews *(n* = 32), to perform the analysis. The quality of the articles included was meticulously evaluated based on the degree of relevance to the topic of this review. A detailed list sorted by phenotypes (Table 1) is included in the following paragraph. The pathogenesis of these syndromes is covered separately by the fourth section of this article.

### 3.2. HHI with Pigment Disorders 

#### 3.2.1. Waardenburg Syndrome (WS)

With an estimated prevalence of 1/42,000, WS is a rare, heterogeneous condition, the features of which include white forelock, depigmented patches of the skin, and SNHI [4,5,97]. These features are characteristic of type 2 WS, while additional clinical symptoms define other types of WS [98]. Patients with type 1 WS present with dystopia canthorum; patients with type 3 WS, a more severe form than type 1 WS, present with dystopia canthorum and musculoskeletal abnormalities of the arms and hands [99,100]. In contrast, patients with type 4 WS present with Hirschsprung disease [101]. 

WS types 2 and 4 can be further classified into subtypes according to the genetic origins. A summary of the subtypes of WS and the genes affected are shown in Table 2. Among the different subtypes of WS, types 2B and 2C are linked to pathogenic variants in unidentified genes mapping to 1p21–p13.3 and 8p23, respectively [98,101,102,103,104,105,106].

WS types 2A and 2 with ocular albinism (WS2-OA) both result from pathogenic variants in the microphthalmia-associated transcription factor gene (*MITF*), and present with SNHI and pigment disorders. WS2-OA also results from pathogenic variants in the *TYR* gene, the main function of the protein product of which is converting tyrosine into melanin [107,108]. Upstream to *MITF*, pathogenic variants in *KITLG* have been found to cause WS type 2 [8,9].

Other pathogenic variants resulting in HHI with pigment disorders include those in *PAX3*, *SOX10*, *EDNRB*, *EDN3*, and *SNAI2* genes. Pathogenic variants in *PAX3* lead to WS types 1 and 3, and those in *SOX10* to WS types 2E and 4C [109]. Patients with a defective *EDNRB* signaling pathway develop either WS types 4Aand 4B, or ABCD syndrome (albinism, black lock of hair, cell migration disorder of gut neurocytes, and sensorineural deafness) [110,111]. Manifestations of these syndromes include Hirschsprung disease, depigmented patches of the skin, white eyelashes, pale blue iridis, and white forelock [103,112]. Homozygous deletions of *SNAI2* have been detected in patients with WS type 2D [106]. 

#### 3.2.2. Tietz Albinism-Deafness Syndrome (TADS)

TADS is a rare autosomal-dominant disease featuring SNHI, generalized pigment loss, and lack of retinal pigmentation [113]. Premature graying of hair during adolescence was observed in a patient [10,107]. Pathogenic variants in *MITF*, including 3-bp del (p.Arg217del), and missense variant c.630C>G (p.Asn210Lys) identified respectively in two families, result in TADS [107,114,115]. Hypopigmentation stems from disrupted transfer of melanosomes from melanocytes to keratinocytes [10]. Although TADS results from alterations in a gene linked to WS type 2, patients do not present with heterochromia or pigmented patches [4,10,114].

#### 3.2.3. COMMAD Syndrome

COMMAD syndrome encompasses coloboma, osteopetrosis, microphthalmia, macrocephaly, albinism, and deafness. Compound heterozygous *MITF* mutations have been detected in two unrelated families with COMMAD syndrome [11]. In contrast to WS type 2A and TADS, which are associated with autosomal-dominant *MITF* mutations, COMMAD syndrome seems to be associated with an autosomal recessive inheritance of MITF, suggesting a crucial role for *MITF* in ocular morphogenesis and bone homeostasis [11].

#### 3.2.4. Histiocytosis-Lymphadenopathy Plus Syndrome

The “histiocytosis-lymphadenopathy plus syndrome” family is a generic term for the H syndrome, Faisalabad histiocytosis (FHC), sinus histiocytosis with massive lymphadenopathy (SHML), and pigmented hypertrichosis with insulin-dependent diabetes mellitus syndrome (PHID) [12]. In the literature, clinical reports and molecular studies are sparse, since it was only recently discovered. The patients had severe SNHI and extensive hyperpigmentation with dark, long hairs. Histologically, polyclonal perivascular lymphohistiocytic infiltrations of the dermis and subcutis were found in hypertrichotic lesions [12,116]. This group of diseases is caused by pathogenic variants in *SLC29A3*, which encodes ENT3, equilibrative nucleoside transporter 3 [12,116,117,118]. This enzyme is in intracellular membranes and mediates cross-membrane nucleoside transportation [119]. Defective ENT3 impairs mitochondrial and lysosomal functions, as well as macrophage homeostasis [12].

#### 3.2.5. Noonan Syndrome with Multiple Lentigines (NSML)

NSML is a rare autosomal-dominant disease without a credible record of global or regional prevalence to date [15]. As its former name “LEOPARD syndrome” indicates, the syndrome features a myriad of clinical manifestations, including multiple lentigines, conduction abnormalities on electrocardiogram, ocular hypertelorism, pulmonic stenosis, abnormal genitalia, retardation of growth, and SNHI [13,15,16]. Pathogenic variants in *PTPN11*, *RAF1*, and *BRAF* genes, encoding parts of the RAS-MAPK (Mitogen-activated protein kinase) signaling cascade, result in NSML types 1, 2, and 3, respectively [14,120,121]. The larger entity, Noonan syndrome (NS), is an autosomal-dominant disease featuring short stature, facial dysmorphia, congenital heart disease, pulmonary valve stenosis, and SNHI, but without multiple lentigines [122]. NS is caused by RAS-MAPK pathway-debilitating variants in *PTPN11*, *RAF1*, *BRAF*, *SOS1*, *KRAS*, among others [122,123].

#### 3.2.6. Vitiligo-Associated Multiple Autoimmune Disease Susceptibility 1 (VAMAS1)

With an unknown prevalence and unclarified mode of inheritance, VAMAS1 features patchy depigmentation of the hair and skin due to the loss of melanocytes, SNHI in certain cases, and a propensity of developing autoimmune thyroid disease, rheumatoid arthritis, and systemic lupus erythematosus [17,124]. The pathogenic variant p.L155H of *NLRP1* has been identified to cause VAMAS1 [125]. *NLRP1* encodes the sensor component of the NLRP1 inflammasome. In response to pathogens, drugs, or damage-associated signals, this protein is recruited, possibly along with PYCARD (PYD And CARD Domain Containing) protein, to assemble the NLRP1 inflammasome and facilitates innate immunity and inflammation [17,126]. Autoimmune response has also been identified in Vogt–Koyanagi–Harada disease (VKHD), another rare multisystem inflammatory disease characterized by pan-uveitis, SNHI, vitiligo, and neurological deficits. However, current studies suggest a melanocyte-specific Th1 cytokine response in VKHD [127,128].

#### 3.2.7. Genophotodermatoses

Xeroderma pigmentosum (XP) and Cockayne syndrome (CS) are autosomal recessive genophotodermatoses resulting from variants in genes involved in DNA repair [22,129,130]. The prevalence of XP is 1/1,000,000 in Europe and the United States (US), and higher in Japan, the Middle East, and North Africa, whereas CS holds a prevalence of 2–3/1,000,000 in the US and Europe [131,132]. 

Photosensitivity, SNHI, and neurologic dysfunction are shared cardinal features of XP and CS [21,22,129,133,134]. Lentiginous macules and poikiloderma are more severe in XP, while loss of subcutaneous orbital fat is distinctive of CS [24,135]. Manifestations of these genophotodermatoses can be attributed to accumulated unrepaired DNA damage following defects in key components of the DNA nucleotide excision repair (NER) pathway. Pathogenic variants in *ERCC6* and *ERCC8* lead to CS types B and A, respectively. Pathogenic variants in *XPA, XPC, RAD2, DDB1, ERCC2, ERCC3, ERCC4, ERCC5*, and *ERCC6* lead to XP groups A-G. Pathogenic variants in *POLH* result in a variant type of XP, which is called XPV [19,20,130,131,134,136].

### 3.3. HHI with Hyperkeratosis

Gap junction-related hyperkeratosis syndromes include palmoplantar keratoderma (PPK) with deafness, Vohwinkel syndrome, Bart-Pumphrey syndrome, hystrix-like ichthyosis with deafness (HID), and keratitis-ichthyosis-deafness syndrome (KID). This is a subgroup of the more generic condition PPKs, but epidemiological studies are lacking due to its rarity [34,137,138,139]. 

SNHI is a shared manifestation among PPK with deafness, Bart-Pumphrey syndrome, HID, KID, and the classic form of Vohwinkel syndrome. By contrast, patients with the variant form of Vohwinkel syndrome do not suffer from SNHI. As for cutaneous manifestation, generalized spiky hyperkeratotic skin is characteristic of HID and KID, while hyperkeratosis is mostly limited to the fingers, palms, and soles in PPK with deafness, Vohwinkel syndrome, and Bart-Pumphrey syndrome [139]. Leukonychia and thickening of the nails have also been reported in cases with Bart-Pumphrey syndrome [31,32,140,141,142].

The five conditions listed in this subgroup share a common genetic cause, i.e., pathogenic variants in *GJB2* [31,143,144,145,146,147,148]. In addition, pathogenic variants in the *GJB6* gene that encodes connexin 30 (Cx30) have also been identified in a family clinically diagnosed with KID [149].

*GJB2* and *GJB6* variants cause both syndromic and non-syndromic HHI. The causal relationship of non-syndromic HHI and pathogenic variants in *GJB2* and *GJB6* have been well-established. Pathogenic variants in *GJB2* serve as the most common cause of autosomal recessive HHI and 20% of non-syndromic hearing loss overall [150,151]. *GJB6* variants are less prevalent than *GJB2* variants but have been identified in 8% of patients with known *GJB2* variants [152]. Whether variants in specific domains of *GJB2* or *GJB6* genes cause syndromic or non-syndromic HHI remains to be elucidated.

### 3.4. Nail Disorders

#### 3.4.1. Autosomal-Dominant Deafness-Onychodystrophy (DDOD) Syndrome

With a prevalence of less than 1/1,000,000, DDOD features severe SNHI, hypoplastic or dystrophic nails, and occasionally, hypoplastic teeth [37,38]. DDOD is associated with pathogenic variants in the *ATP6V1B2* gene [39,40].

#### 3.4.2. Deafness, Onychodystrophy, Osteodystrophy, Mental Retardation, and Seizures (DOORS) Syndrome

With an estimated prevalence of less than 1/1,000,000, the autosomal recessively inherited DOORS differs from DDOD regarding neurological symptoms, including mental retardation and seizures [41,153,154,155]. Pathogenic variants in *TBC1D24* are the causative genetic alterations associated with DOORS [42,43,156,157,158,159].

#### 3.4.3. Heimler Syndrome and Other Peroxisomal Biogenesis Disorders (PBDs)

PBDs are a spectrum of autosomal recessive disorders of different severity, of which Zellweger syndrome (ZS) is the most severe form; neonatal adrenoleukodystrophy (NALD) presents with milder symptoms, and infantile Refsum disease (IRD) and Heimler syndrome constitute the mildest forms. The prevalence of PBDs is 1/50,000 and 1/500,000 in North America and Japan, respectively, while epidemiological figures on Heimler syndrome are to be determined [51,160,161]. PBDs result from pathogenic variants in peroxin-encoding genes, i.e., *PEX1, PEX2, PEX3, PEX5, PEX6, PEX10, PEX11β, PEX12, PEX13, PEX14, PEX16, PEX19,* and *PEX26* [50]. Heimler syndromes 1 and 2 are at the mildest end of the PBD spectrum, and are caused by pathogenic variants in *PEX1* and *PEX6*, respectively [47,51,54]. Errors in the production of peroxins result in impaired myelin sheath formation and neurological deficits, including neonatal seizures, hypotonia, and developmental delays. Decreased peroxisome functionality in the liver and kidneys gives rise to the associated symptoms, including hepatomegaly, intrahepatic biliary dysgenesis, and hydronephrosis. SNHI and distinctive craniofacial features are also cardinal features of PBDs. Nail abnormalities, including Beau lines and leukonychia, have been reported in patients with Heimler syndrome [46,51,53,54,55]. 

#### 3.4.4. Nail-Patella Syndrome (NPS)

NPS is an autosomal dominantly inherited syndrome with a prevalence of 1/50,000 live births. Nail dysplasia is the cardinal dermatologic manifestation of NPS. Nail changes include partially exposed and/or narrow nail beds, median or partial median clefts, dystrophic nail surfaces, and absence of nails. Fifth finger clinodactyly, hyperextensibility of the proximal interphalangeal joint, loss of creases over the distal interphalangeal joint and triangular lunulae have been reported in NPS patients. Other key features include malformation of dorsal mesenchyme-derivatives, including muscles, tendons, and the patella, along with ocular or renal involvement [162,163,164,165]. Hearing loss has also been reported in patients with NPS [59]. Genetically, pathogenic variants in *LMX1B* are considered to be causative of NPS [57,59,166,167].

#### 3.4.5. Nephropathy with Pretibial Epidermolysis Bullosa and Deafness (NPEBD)

The only three cases with NPEBD feature nail dystrophy, blisters in the lower extremities, SNHI, and proteinuria in the nephrotic range [65]. Single-nucleotide insertion (383_384insG) in *CD151*, a gene encoding a component of hemidesmosomes, has been found in all cases. This result implies a role for *CD151* in the maintenance of the normal structure and function of the skin, inner ear, and the glomeruli and tubules in the kidney [62,63,64].

### 3.5. HHI with Connective Tissue Disorders

#### 3.5.1. Hyperelasticity of the Skin, Excess Skin, or Hypermobility of the Joints 

Brittle cornea syndrome (BCS), Ehlers-Danlos syndrome musculocontractural type 1 (EDSMC1), congenital symmetric circumferential skin creases (CSCSC) types 1 and 2, and microphthalmia with linear skin defects syndrome (MLS) are connective tissue disorders that present with distinct cutaneous findings and hearing impairment. Epidemiological data are scant due to the rarity of these conditions. 

BCS1 and BCS2 are characterized by hyperelasticity of the skin, hypermobility of the joints, blue sclerae, keratoconus, and keratoglobus. Mixed conductive and sensorineural hearing impairments have been reported in cases of BCS, with frequent manifestations that are milder and of later onset than the ophthalmic symptoms. BCS1 and BCS2 result from pathogenic variants in *ZNF469* and *PRDM5*, respectively [66,69,168,169,170,171]. 

EDSMC1 is characterized by dysmorphisms throughout the musculoskeletal system, easy bruisability, joint hypermobility, and hearing impairment, in certain cases. EDSMC1 can be attributed to pathogenic variants in *CHST14* [67,72,74,172,173].

Patients with CSCSC1 and CSCSC2 feature excess skin and ringed creases, as well as hearing impairment [77,174]. CSCSC is considered a tubulinopathy. Accordingly, pathogenic variants in *TUBB* and *MAPRE2* are the causative genetic alterations associated with CSCSC1 and CSCSC2, respectively [77,79,174].

Microphthalmia with linear skin defects syndrome (MLS), or linear skin defects with multiple congenital anomalies 1 (LSDMCA1), also features linear skin defects and hearing impairment, and is caused by pathogenic variants in the holocytochrome c-type synthase-encoding *HCCS* gene [175,176,177,178,179].

#### 3.5.2. Cryopyrin-Related Autoinflammatory Syndromes (CAPS)

A spectrum of autosomal-dominant autoinflammatory syndromes of different severities, including Muckle-Wells syndrome (MWS), familial cold autoinflammatory syndromes 1 and 2 (FCAS1, FCAS2), and chronic infantile neurologic cutaneous and articular (CINCA) syndrome, are related to cryopyrin. The prevalence of CAPS in France and the USA is estimated to be 1/360,000 and 1–2/1,000,000 individuals, respectively [180,181]. Patients present with urticaria, rash, or limb swelling, aggravated by cold temperature [182,183]. SNHI may result from inflammatory processes in the cochlea [180,184,185,186]. FCAS2 arises from pathogenic variants in *NLRP12*, while MWS, FCAS1, and CINCA syndrome can be attributed to gain-of-function pathogenic variants in the *NLRP3* gene [90,185].

### 3.6. Others

Cornelia de Lange syndrome, with an overall prevalence of 1.6–2.2/100,000, is a mostly sporadic condition characterized by multiple organ-system defects [187]. Patients present with dysmorphic face and upper extremities, and growth and mental retardation. Hearing impairment, either sensorineural or conductive, is nearly ubiquitous [96,188,189,190]. The skin is mostly spared, but cavernous hemangiomas have been observed in a case with Cornelia de Lange syndrome 1 [190,191,192,193,194]. *NIPBL, SMC1A, SMC3, RAD21,* and *HDAC8* are the five genes associated with Cornelia de Lange syndrome [191,192,193,194,195].

### 3.7. Frequency of SNHI in HHI with Cutaneous Abnormalities

The frequency of SNHI differs among various syndromic HHI with cutaneous abnormalities. For instance, SNHI has been found in over 70% of cases with WS, TADS, COMMAD, or NSML syndromes [11,15,97,114]; and in approximately half of patients with other syndromes such as histiocytosis-lymphadenopathy plus syndrome [196]. On the contrary, the frequency of SNHI is difficult to estimate in rarer conditions such as NPS, DOORS, or DDOD.

## 4. Molecular Mechanisms Underlying Various Types of HHI with Cutaneous Abnormalities

The pathogenesis behind some of the syndromes discussed in the present study has been documented in the literature. Generally, cutaneous manifestations associated with HHI can be classified into three categories: pigment, hyperkeratosis/nail, and connective tissue disorders (Table 3). We herein summarize the molecular mechanisms underlying syndromic HHI with different cutaneous involvements.

### 4.1. HHI with Pigment Disorders

Syndromic HHI with pigmentary disorders was found associated with diverse molecular mechanisms, including differentiation and migration of melanocytes, RAS-MAPK signaling, and DNA repair.

#### 4.1.1. Differentiation and Migration of Melanocytes

As mentioned above, certain subtypes of WS type 2, TADS, and COMMAD syndrome can be attributed to pathogenic variants in *MITF*, while other types of WS are linked to pathogenic variants in *PAX3, SNAI2, SOX10, EDNRB, EDN3,* and *KITLG*. These genes are crucial for the differentiation and migration of melanocytes.

The *MITF* gene on chromosome 3p14.1–p12.3 encodes the protein MITF, which is a basic helix-loop-helix (hHLH)-leucine zipper and plays a role in the development of various cell types, including neural crest-derived melanocytes, optic cup-derived retinal pigment epithelial cells, and melanocytes [199]. In melanocyte differentiation, MITF transactivates the promoter activity of the tyrosinase gene *TYR* [200,201,202,203]. Thus, pathogenic variants in the *MITF* gene might lead to absence of melanocytes in the skin, hair, eyes, and stria vascularis of the cochlea. *PAX3* and *SOX10* encode transcription factors that synergistically regulate the expression of *MITF*, and pathogenic variants in these two genes also result in pigmentary abnormalities of the hair, skin, and eyes, as well as in SNHI. Specifically, *SOX10* activates the MITF pathway by binding onto the *MITF* promoter. Loss-of-function variants including a 1076delGA in exon 5, a 6-bp insertion in exon 4, along with a tyr83-to-ter variant and a glu189-to-ter variant were found to cause WS type 4C [101]. On the other hand, a ser135-to-thr variant was identified in a patient with WS type 2E [109]. The activation of KITLG-KIT signaling pathway leads to the activation of downstream *MITF*, and defective *KITLG* has been linked to WS type 2 [8,9].

Pathogenic variants in *EDNRB*, the gene encoding the endothelin-B receptor, and those in the gene for its ligand endothelin-3 (EDN3) also result in a lack of melanocytes. *EDNRB* and *EDN3* take part in the migration and proliferation of neural crest-derived cells including melanocytes [204]. *SNAI2* encodes a zinc finger protein essential to the development of neural crest-derived cells [205]. A pathogenic variant in *Slugh*, the murine homolog of the human *SNAI2* gene, causes pigmentary disorders in mice including white forelock and patchy depigmentation over the ventral body, tail, and feet. Hyperactivity and circling behavior observed in *Slugh*-deficient mice implied the presence of auditory and vestibular dysfunctions. These findings implicate a role for *SNAI2* in the development and/or migration of neural crest-derived cells [98,106].

#### 4.1.2. RAS-MAPK Signaling 

NSML types 1, 2, and 3 result from pathogenic variants in *PTPN11*, *RAF1*, and *BRAF* genes, respectively, products of which all participate in the RAS-MAPK signaling cascade. The tyrosine phosphatase encoded by *PTPN11* relays signals from cell membrane receptors to cytoplasmic tyrosine kinases and up-regulates the MAPK signaling pathway [206]. The serine/threonine-protein kinase encoded by *RAF1* links Ras GTPases to the MAPK/ERK (extracellular signal-regulated kinases) cascade and serves as a decision point leading cells to proliferate, differentiate, or undergo apoptosis. The serine/threonine-protein kinase B-raf, encoded by *BRAF*, facilitates cell membrane-nucleus signaling through phosphorylation of MAP2K1 [207,208]. It may further contribute to postsynaptic responses of hippocampal neurons [209].

Histological specimens of lentiginous lesions of NSML cases with pathogenic *PTPN11* variants revealed increased numbers of melanocytes and pigments throughout the epidermis, while immunohistochemical studies revealed increased expression levels of endothelin-1 (ET-1), phosphorylated Akt, mTOR, and STAT3 in lentiginous epidermis compared with non-lentiginous skin areas. Higher melanin synthesis rates of human melanoma cells expressing tyrosine-protein phosphatase non-receptor type 11 have been observed in vitro, supporting the link between *PTPN11* and hyperpigmentation in NSML patients [210]. Vestibulocochlear anomalies and atrophic cochlear neurons have been observed in patients with pathogenic *PTPN11* variants [211]. 

#### 4.1.3. DNA Repair

XP and CS are caused by defective DNA repair pathways. Defects in *XPC* and *XPE,* factors in charge of global genome nucleotide excision repair (GG-NER), in *XPA, XPG, XPB,* and *XPD*, which oversee DNA unwinding, as well as in *XPF* and *XPG*, mediating excision of the damaged nucleotides, lead to hyper- and hypopigmented macules in sun-exposed areas and an increased risk of skin malignancies [212]. Defects in *POLH* lead to XPV, a rare subtype of XP. 

Increased numbers of melanocytes and elevated melanin levels have been found in skin specimens of freckles from XPC patients. Hyperpigmentation in XP results from increased proliferation and early differentiation of melanocytes due to the mutagenic tendency of cells with impaired GG-NER [21]. UV(ultraviolet)-induced oxidative stress could also induce hyperpigmentation. Melanogenesis is regulated through the ERK signaling pathway activated by mitochondrial reactive oxidative species [213]. The production of UV-induced protective pigments is up-regulated by the mitochondrial protein prohibitin [214,215]. Defective repair mechanisms and UV-induced changes in microenvironment spark apoptotic pathways in XP melanocytes, resulting in hypopigmented areas. Apoptosis of cells in XP patients is triggered by lower doses of UV than needed to induce apoptosis in normal cells [216,217,218,219]. Compared to XP, the phenotype of CS includes progeroid appearance, generally without pigmentary changes [220]. XP and CS are associated with SNHI of cochlear origin on audiological assessments. Temporal bone histology at autopsy revealed atrophy of the sensory epithelium and neurons in the cochlea. Atrophies of the stria vascularis, hair cells, or Scarpa’s ganglion have been observed in different cases of XP [133,221]. 

### 4.2. HHI with Hyperkeratosis Or Nail Disorders

#### 4.2.1. HHI with Hyperkeratosis

Syndromic HHI with hyperkeratosis are caused by pathogenic variants in two gap junction genes, *GJB2* and *GJB6*, which encode connexins that are key to intercellular signaling [222]. The ectoderm-derived epithelia of the inner ear and the epidermis share the expression of Cx26 and Cx30 [223,224]. In the skin, Cx26 is mainly expressed in the palmoplantar epidermis and the inner and outer root sheaths of the human hair follicle, while Cx30 is predominantly expressed in the differentiated layers of the interfollicular epidermis [225,226,227]. Defective connexins result in leaky hemichannels and impaired intercellular communication [139,228]. Cx26 plays a role in wound healing and is also involved in the normal differentiation and proliferation of keratinocytes, which may explain the hyperkeratosis observed in individuals with defective Cx26 [228,229]. 

In the inner ear, connexins are abundantly expressed in the cochlear sensory epithelium, and are key factors in maintaining the potassium levels of the endolymph [20]. Immunochemical stainings have revealed that Cx26 and Cx30 are expressed in the spiral limbus, spiral ligament, stria vascularis, and supporting cells of the organ of Corti. Cx26 contributes to normal development of the cochlear sensory epithelium, and compromised inositol 1,4,5-trisphosphate (Ins(1,4,5)P3) permeability of Cx26 has been implicated as a cause of SNHI [230,231]. Additionally, the endocochlear potential generated by the stria vascularis is remarkably disturbed in Cx30-deficient mice [232]. 

*GJB4* encodes Cx30.3, pathogenic variants in which have been linked to erythrokeratodermia variabilis et progressiva, or EKVP [233]. EKVP is a rare, mostly autosomal-dominant genodermatosis featuring erythema gyratum repens and stable hyperkeratotic plaques [234]. How *GJB4* variants induce EKVP remains hypothetical. The link between *GJB4* and SNHI has not yet been well-established either; however, *GJB4* variants have been identified in 11 patients with non-syndromic hearing loss in Taiwan. These patients suffered from congenital bilateral SNHI but no skin lesion was found [235,236]. *GJB4* variants have also been identified in Iranian patients with autosomal recessive non-syndromic hearing loss [237,238]. These pilot genotype-phenotype correlation studies serve as the steppingstone to clarify the link between *GJB4* and SNHI.

#### 4.2.2. HHI with Nail Disorders

The molecular underpinnings of syndromic HHI with nail disorders involve a plethora of genes related to proton transportation, vesicle transportation, peroxisome function, and hemidesmosomes.

The DDOD-linked *ATP6V1B2* gene encodes a component of the vacuolar ATPase for proton transportation. Impaired lysosomal acidification due to V-ATPase deficiency undermines the Wnt signaling pathway, which is important for normal limb organogenesis. This may explain the dystrophic or atrophic nails present in DDOD patients [239,240,241]. Immunostaining of mouse cochlea showed predominant expression of *Atp6v1b2* in the organ of Corti and spiral ganglion neurons. Consistent with histological findings, auditory brainstem response tests showed elevated hearing thresholds in cochlea-specific *Atp6v1b2*-knockdown mice, supporting the link between *ATP6V1B2* and SNHI [39]. 

The DOORS-linked *TBC1D24* encodes a GTPase-activating protein crucial to vesicle transportation [242,243]. *TBC1D24* regulates migration of neural crest cells in coordination with ephrinB2 and the scaffold protein Dishevelled (Dsh) [244]. Immunostaining of mouse cochlea showed predominant expression of *Tbc1d24* in inner and outer hair cells, and weaker expression in spiral ganglion neurons [245]. Nails and membranous labyrinth are both ectoderm-derived, which underlies the coexistence of nail dystrophy and SNHI [155].

Heimler syndromes 1 and 2 arise from pathogenic variants in *PEX1* and *PEX6*, respectively, which lead to impaired peroxisome biogenesis [49,52]. Decreased metabolism of very long chain fatty acids underpins the cutaneous findings in the PBD spectrum [45,48]. Reduced or defective peroxisomes in Heimler syndrome patients have been found through immunofluorescence microscopy [51,246]. As oxidative stress is linked to hearing loss, this finding consolidates the relationship between peroxisomal dysfunction and SNHI in Heimler syndrome [49,247,248].

The NPS-related gene *LMX1B* encodes the LIM homeobox transcription factor, defects in which hinder limb and skin development; the dystrophic nails and orthopedic abnormalities may result from altered embryonic dorsoventral patterning [58,60,61]. Strong expression of the mouse homolog *Lmx1b* in the hindbrain implies that *LMX1B* variants disturb inner ear development [249].

The NPEBD-linked *CD151* encodes a tetraspan protein crucial to hemidesmosome integrity [63]. *CD151* facilitates basement membrane formation, migration of keratinocytes, and adhesion and migration of epithelial cells, highlighting its role in skin integrity and wound healing [250]. Hearing loss has been observed in laminin-deficient mice. As *CD151* is key to laminin-binding among other tetraspanin-integrin interactions, defective *CD151* may impair normal hearing [251,252].

### 4.3. HHI with Connective Tissue Disorders

Syndromic HHI with connective tissue disorders result from the deregulation of the extracellular matrix (ECM), dermatan-sulfate (DS) biosynthesis, microtubule assembly, mitochondria-mediated cell death, and inflammatory cascades.

The products of BCS1 and BCS2-associated genes, i.e., zinc finger protein 469 encoded by *ZNF469*, and PR domain-containing protein 5 encoded by *PRDM5*, regulate and maintain the ECM [169,253]. Pathogenic variants in *PRDM5* lead to decreased or disorganized vital ECM components, including collagen I fibers and decorin, which has been shown in patient-derived fibroblast models [253,254]. Disorganized ECM leads to skin fragility and hyperelasticity in BCS patients [171]. SNHI has been documented in both *PRDM5*- and *ZNF469*-associated types of BCS [169,253]. 

The enzyme products of EDSMC1 and EDSMC2-causing genes *CHST14* and dermatan-sulfate epimerase (*DSE*) are dermatan-4-sulfotransferase-1 (D4ST1) and dermatan-sulfate epimerase, respectively. These enzymes facilitate DS biosynthesis [173,255]. D4ST1 dysfunction hinders normal production and assembly of the ECM. Additionally, disrupted ECM components, including fibronectin and fibrillar collagen types I, III, and V, have been found in D4ST1-deficient patients [74,173]. These ECM defects lead to skin hyperextensibility, easy bruising, increased palmar wrinkling, and propensity to subcutaneous hematoma formation in EDSMC patients [71,173]. EDSMC1 patients with high-tone SNHI have been reported in the literature [72,173]. EDSMC2-causing variants in *DSE* also result in dysfunctional DS and ECM disarray; however, SNHI has not been reported in EDSMC2 patients [256].

Products of CSCSC1 and CSCSC2-associated genes, i.e., tubulin β chain encoded by *TUBB* and end-binding protein 2 encoded by *MAPRE2*, are crucial to microtubule assembly and polymerization [77,78]. Altered *MAPRE2* expression perturbs branchial arch pattering, explaining the skin and craniofacial anomalies in CSCSC1 patients [77]. In cochlear sensory cells, microtubules form both dynamic and supporting structures of the organ of Corti [257]. Immunohistochemical staining of the inner ear revealed diffuse expression of β-tubulin, an autoantigen targeted in autoimmune inner ear disease [258,259,260,261,262,263,264]. Antibodies recognizing β-tubulin were isolated in the serum of 59% of patients with Meniere’s disease [265]. Taken together, microtubule assembly and dynamics are crucial for maintaining normal hearing.

The product of the MLS gene *HCCS* is crucial to mitochondrial-mediated apoptosis [175,176,177]. Defects in this synthase results in a shift from apoptosis to necrosis and induces inflammation and damage to neighboring cells, inducing the cutaneous manifestation of MLS [266]. 

The CAPS-linked *NLRP3* and *NLPR12* are mainly expressed in neutrophils and chondrocytes, and gain-of-function variants lead to over-activation of the inflammasome, overstimulation of interleukin (IL)-1β receptors, and overproduction and secretion of IL-1β [185,267,268]. Following the constitutive activation of the *NLRP3* inflammasome, mast cells in CAPS patients produce IL-1β, induce neutrophil migration, and promote vascular leakage independent of stimuli [269]. Tissue-resident macrophage/monocyte-like cells reside perivascularly throughout the cochlea [185,270]. *NLRP3* inflammasome-induced secretion of IL-1β induces cochlear inflammation, and thus SNHI [271,272]. The recombinant IL-1 receptor antagonist (IL-1Ra) Anakinra ameliorates SNHI, consolidating the role of IL-1β in hearing loss [185,268]. IL-1β also causes higher permeability of cytokines between the perilymph and CSF (cerebrospinal fluid) space via the modiolus, prompting spiral ligament fibrocytes to produce inflammatory mediators [182]. 

## 5. Conclusions

Listed in this review is a comprehensive array of syndromic HHI with abnormal cutaneous findings. This provides an outline for clinicians and researchers encountering patients with abnormal manifestations, which are evident in the setting of an outpatient clinic appointment (e.g., in a well-baby clinic). The pathogenesis of the skin manifestations and syndromic HHI of certain syndromes has not yet been fully elucidated. Further molecular and functional studies are necessary to unveil the underlying mechanisms.

## Figures and Tables

**Table 1:** Summary of syndromic hereditary hearing impairment (HHI) with cutaneous abnormalities.

Syndrome	Genes Involved	OMIM Number	Mode of Inheritance	Clinical Findings Other Than SNHI	Ref.
**Pigment disorders**					
Waardenburg syndrome type 1	*PAX3*	193500	AD	Pigmentary abnormalities of the hair, skin, and eyes, dystopia canthorum	[4,5,6]
Waardenburg syndrome type 2	*MITF, SNAI2, SOX10, KITLG*	184745, 193510, 600193, 606662, 608890, 611584	AD, AR	Pigmentary abnormalities of the hair, skin, and eyes	[4,5,7,8,9]
Waardenburg syndrome type 3	*PAX3*	148820	AD, AR	Pigmentary abnormalities of the hair, skin, and eyes, dystopia canthorum, upper limb abnormalities	[4,5]
Waardenburg syndrome type 4	*EDNRB, EDN3, SOX10*	277580, 613265, 613266	AD, AR	Pigmentary abnormalities of the hair, skin, and eyes, Hirschsprung disease	[4,5]
Tietz albinism-deafness syndrome	*MITF*	103500	AD	Albinism, lack of retinal pigmentation, absent eyebrows	[10]
COMMAD syndrome	*MITF*	617306	AR	Microphthalmia, coloboma, cranial dysmorphism, cataract, osteopetrosis, pigmentary abnormalities of the hair, skin, and eyes	[11]
Histiocytosis-lymphadenopathy plus syndrome	*SLC29A3*	602782	AR	Hyperpigmentation, hypertrichosis, lymphadenopathy, hepatosplenomegaly, heart anomalies, and hypogonadism	[12]
Noonan syndrome with multiple lentigines	*PTPN11, RAF1, BRAF*	151100, 611554, 613707	AD	Multiple lentigines, ocular hypertelorism, growth retardation, electrocardiographic conduction abnormalities, pulmonary stenosis, abnormal genitalia	[13,14,15,16]
Vitiligo-associated multiple autoimmune disease susceptibility	*NLRP1*	606579	unknown	Patchy depigmentation of the skin and hair, elevated risk of autoimmune diseases	[17]
Xeroderma pigmentosum	*XPA, XPC, DDB2 (XPE), ERCC2 (XPD), ERCC3 (XPB), ERCC4 (XPF), ERCC5 (XPG)*, *ERCC6 (CSB), POLH (XPV)*	610651, 278760, 278780, 278750	AR	Cutaneous photosensitivity, microphthalmia, cataracts, optic atrophy, pigmentary retinal degeneration, neurological impairments, growth defects	[18,19,20,21]
Cockayne syndrome	*ERCC6, ERCC8 (CSA)*	133540, 216400	AR	Cutaneous photosensitivity, thin and dry hair, pigmentary retinopathy, dental caries, progeroid appearance, characteristic stance in ambulatory patients	[22,23,24,25]
**Hyperkeratoses**					
Palmoplantar keratoderma with deafness	*GJB2*	148350	AD	Hyperkeratosis of the palms and soles	[26,27]
Vohwinkel syndrome	*GJB2*	124500	AD	Palmoplantar hyperkeratosis, epidermal thickening of the knuckles and knees, pseudoainhum or autoamputation of the fingers and toes	[28,29,30]
Bart-Pumphrey syndrome	*GJB2*	149200	AD	Palmoplantar hyperkeratosis, knuckle pads, leukonychia	[31,32,33]
Hystrix-like ichthyosis with deafness	*GJB2*	602540	AD	Erythroderma, hyperkeratosis, hypotrichosis of eyebrows, eyelids, and scalp	[34,35]
Keratitis-ichthyosis-deafness syndrome	*GJB2, GJB6*	148210	AD	Keratopachydermia and constrictions of the fingers and toes, loss of eyebrows and eyelashes	[34,36]
**Nail disorders**					
Autosomal-dominant deafness-onychodystrophy syndrome	*ATP6V1B2*	124480	AD	Dystrophic or hypoplastic nails, syndactyly, triphalangeal thumbs, tooth agenesis	[37,38,39,40]
Deafness, onychodystrophy, osteodystrophy, mental retardation, and seizures syndrome	*TBC1D24*	220500	AR	Dystrophic or hypoplastic nails, syndactyly, triphalangeal thumbs, tooth agenesis, mental retardation, seizures	[41,42,43,44]
Heimler syndrome 1	*PEX1*	234580	AR	Beau lines, enamel hypoplasia in the permanent dentition	[45,46,47,48,49,50,51,52,53,54]
Heimler syndrome 2	*PEX6*	616617	AR	Beau lines, enamel hypoplasia in the permanent dentition	[45,47,48,49,50,51,52,53,54,55]
Nail-patella syndrome	*LMX1B*	161200	AD	Dysplastic or hypoplastic nails, absent or hypoplastic patellae, iliac horns, abnormality of the elbows interfering with pronation and supination, nephropathy	[56,57,58,59,60,61]
Nephropathy with pretibial epidermolysis bullosa and deafness	*CD151*	609057	unknown	Multiple, recurrent, infected skin blisters of the legs, followed by atrophy, nail dystrophy, bilateral lacrimal duct stenosis, proteinuria in the nephrotic range	[62,63,64,65]
**Connective tissue disorders**					
Brittle cornea syndrome 1	*ZNF469*	229200	AR	Hyperelasticity of the skin, hypermobility of the joints, blue sclerae, keratoconus, keratoglobus	[66,67,68]
Brittle cornea syndrome 2	*PRDM5*	614170	AR	Hyperelasticity of the skin, hypermobility of the joints, blue sclerae, keratoconus, keratoglobus	[66,67,69,70]
Ehlers-Danlos syndrome musculocontractural type 1	*CHST14*	601776	AR	Hyperextensibility and fragility of the skin, hypermobility of joints, cranial dysmorphism, contracture of the thumbs and fingers, adducted thumb, clubfoot, kyphoscoliosis	[71,72,73,74,75]
Congenital symmetric circumferential skin creases type 1	*TUBB*	156610	AD	Excess skin, ringed creases of the limbs, hypertrichosis, mental retardation, facial dysmorphism, neurological abnormalities	[76,77]
Congenital symmetric circumferential skin creases type 2	*MAPRE2*	616734	AD	Excess skin, ringed creases of the limbs, hypertrichosis, mental retardation, facial dysmorphism, neurological abnormalities	[77,78,79]
Microphthalmia with linear skin defects syndrome	*HCCS*	309801	XLD	Irregular linear areas of erythematous skin hypoplasia, microphthalmia, short stature, corneal opacities, developmental delay, agenesis of the corpus callosum	[80,81,82,83]
Familial cold autoinflammatory syndrome 1	*NLRP3*	120100	AD	Episodic urticarial rash and swelling of the extremities after exposure to cold	[84,85]
Familial cold autoinflammatory syndrome 2	*NLRP12*	611762	AD	Episodic urticarial rash, fever, headache, lymphadenopathy, arthralgia, and myalgia after exposure to cold	[86,87,88]
Muckle-Wells syndrome	*NLRP3*	191900	AD	Episodic rash, fever, arthralgia, and renal amyloidosis	[89,90,91]
Chronic infantile neurologic cutaneous and articular syndrome	*NLRP3*	607115	AD	Persistent and migratory urticarial rash, progressive visual defect and neurologic impairment, and joint abnormalities	[92,93,94,95]
**Others**					
Cornelia de Lange syndrome	*NIPBL, SMC1A, SMC3, RAD21, HDAC8*	122470, 300590, 610759, 614701, 300882	AD, XLD	Hemangioma, facial dysmorphisms, including hypertrichosis, synophrys, and bushy eyebrows	[96]

SNHI: sensorineural hearing loss. AD: autosomal dominant. AR: autosomal recessive. XLD: X-linked dominant.

**Table 2:** Subtypes of Waardenburg syndrome (WS) and affected genes.

Subtypes of WS	Affected Genes	Locations
Type 1	*PAX3*	2q36.1
Type 2A	*MITF*	3p13
Type 2A with ocular albinism	*MITF, TYR*	3p13, 11q14.3
Type 2B	*-*	1p21–p13.3
Type 2C	*-*	8p23
Type 2D	*SNAI2*	8q11.21
Type 2E	*SOX10*	22q13.1
Type 2, subtype not designated	*KITLG*	12q21.32
Type 3	*PAX3*	2q36.1
Type 4A	*EDNRB*	13q22.3
Type 4B	*EDN3*	20q13.32
Type 4C	*SOX10*	22q13.1

**Table 3:** Molecular mechanisms underlying syndromic hereditary hearing impairment (HHI) with cutaneous abnormalities and expression of the affected genes in the inner ear and epidermis.

Affected Molecular Pathways	Phenotype	Gene Symbol	Fold Change (Hair Cell/Non-Hair Cell) in the Inner ear ^1^	Main Expressors in the Epidermis ^2^
**HHI with pigment disorders**				
MITF-related	Waardenburg syndrome	*MITF*	0.17	K, M
		*PAX3*	0.15	K, L, M
		*SOX10*	0.21	M
Non-MITF-related	Waardenburg syndrome	*EDNRB*	0.08	low expression in K, M
		*EDN3*	0.09	no data
		*SNAI2*	0.14	F, K, L, M
RAS-MAPK signaling	Noonan syndrome with multiple lentigines	*PTPN11*	1.27	diffuse in epidermal cells
		*RAF1*	1.35	low expression
		*BRAF*	2.77	K, L, M
DNA repair	Xeroderma pigmentosum	*XPA*	3.00	K, L, M
		*XPC*	1.06	K, M
		*DDB2 (XPE)*	1.34	K, L, M
		*ERCC2 (XPD)*	1.05	K, L, M
		*ERCC3 (XPB)*	0.84	K, L, M
		*ERCC4 (XPF)*	1.57	K
		*ERCC5 (XPG)*	1.03	K, L
		*ERCC6 (CSB)*	6.04	no data
		*POLH (XPV)*	1.09	K, L, M
	Cockayne syndrome	*ERCC6 (CSB)*	6.04	no data
		*ERCC8 (CSA)*	1.09	no data
Intracellular cross-membrane transportation	Histiocytosis-Lymphadenopathy plus syndrome	*SLC29A3*	1.41	not detected
Inflammasome assembly	Vitiligo-associated multiple autoimmune disease susceptibility	*NLRP1*	*nlrp1a*: 0.43 *nlrp1b*: 4.03	K, L
**HHI with hyperkeratosis or nail disorders**				
Gap junctions	Palmoplantar keratoderma with deafness, Vohwinkel syndrome, Bart-Pumphrey syndrome, hystrix-like ichthyosis-deafness syndrome, keratitis-ichthyosis-deafness syndrome	*GJB2*	0.31	K, L, M
	Keratitis-ichthyosis-deafness syndrome	*GJB6*	0.10	no data
Vacuolar proton transportation	Dominant deafness-onychodystrophy	*ATP6V1B2*	2.97	K, L, M
Transportation of vesicles	Deafness, onychodystrophy, osteodystrophy, mental retardation, and seizures	*TBC1D24*	0.75	not detected
Peroxisome biogenesis	Heimler syndrome 1	*PEX1*	1.62	not detected
	Heimler syndrome 2	*PEX6*	0.86	low expression
LIM homeobox-dependent transcription	Nail-patella syndrome	*LMX1B*	3.04	no data
Formation and maintenance of hemidesmosomes	Nephropathy with pretibial epidermolysis bullosa and deafness	*CD151*	0.65	K, M
**HHI with connective tissue disease**				
Extracellular matrix regulation	Brittle cornea syndrome	*ZNF469*	No data	no data
		*PRDM5*	0.55	K, L, M
	Ehlers-Danlos syndrome, musculocontractural type 1	*CHST14*	0.22	not detected
Microtubule dimerization and dynamics	Congenital symmetric circumferential skin creases 1	*TUBB*	*tubb1*: 1.49 *tubb2a*: 1.24 *tubb2b*: 3.46 *tubb3*: 22.03 *tubb4a*: 0.57 *tubb4b*: 3.30 *tubb5*: 0.62 *tubb6*: 0.43	F, M
	Congenital symmetric circumferential skin creases 2	*MAPRE2*	0.85	low expression in F, K, M
Oxidative phosphorylation and apoptosis	Microphthalmia with linear skin defects syndrome	*HCCS*	0.63	low expression in F, L, K, M
Inflammasome assembly	Cryopyrin-associated periodic syndrome	*NLRP3*	0.05	K, M
		*NLRP12*	No data	L, M

^1^ Fold change (hair cell/non-hair cell) denotes the ratio of hair cells to non-hair cells in mouse utricle and cochlea derived by fluorescence-activated cell sorting (FACS) in the SHIELD (Shared Harvard Inner-Ear Laboratory Database) database [197]. ^2^ Protein expression of the affected genes in the epidermis as referenced from The Human Protein Atlas (http://www.proteinatlas.org) [198] Abbreviations: F, fibroblasts; K, keratinocytes; L, Langerhans cells; M, melanocytes.
